# The Impact of Anthocyanins and Iridoids on Transcription Factors Crucial for Lipid and Cholesterol Homeostasis

**DOI:** 10.3390/ijms22116074

**Published:** 2021-06-04

**Authors:** Maciej Danielewski, Agnieszka Matuszewska, Adam Szeląg, Tomasz Sozański

**Affiliations:** Department of Pharmacology, Wroclaw Medical University, Jana Mikulicza-Radeckiego 2, 50-345 Wroclaw, Poland; agnieszka.matuszewska@umed.wroc.pl (A.M.); adam.szelag@umed.wroc.pl (A.S.); tomasz.sozanski@umed.wroc.pl (T.S.)

**Keywords:** anthocyanins, iridoids, transcription factors, lipids, cholesterol

## Abstract

Nutrition determines our health, both directly and indirectly. Consumed foods affect the functioning of individual organs as well as entire systems, e.g., the cardiovascular system. There are many different diets, but universal guidelines for proper nutrition are provided in the WHO healthy eating pyramid. According to the latest version, plant products should form the basis of our diet. Many groups of plant compounds with a beneficial effect on human health have been described. Such groups include anthocyanins and iridoids, for which it has been proven that their consumption may lead to, inter alia, antioxidant, cholesterol and lipid-lowering, anti-obesity and anti-diabetic effects. Transcription factors directly affect a number of parameters of cell functions and cellular metabolism. In the context of lipid and cholesterol metabolism, five particularly important transcription factors can be distinguished: liver X receptor (LXR), peroxisome proliferator-activated receptor-α (PPAR-α), peroxisome proliferator-activated receptor-γ (PPAR-γ), CCAAT/enhancer binding protein α (C/EBPα) and sterol regulatory element-binding protein 1c (SREBP-1c). Both anthocyanins and iridoids may alter the expression of these transcription factors. The aim of this review is to collect and systematize knowledge about the impact of anthocyanins and iridoids on transcription factors crucial for lipid and cholesterol homeostasis.

## 1. Introduction

It is a truism to say that nutrition directly reflects our health. Many scientific publications have proven the beneficial effects of a proper diet on, among others, blood parameters, skin condition or the work of key metabolic organs such as the pancreas, liver or kidneys. Moreover, although, globally, store shelves are filled with highly processed, heavily sweetened and high-saturated-fat products, natural and organic ingredients are becoming more and more popular. Various campaigns are organized—sometimes national or nationwide—calling for populations to adopt an appropriate dietary regimen. Furthermore, the mass media, celebrities and public figures often promote a healthy lifestyle, frequently by setting an example.

Each individual has their own unique dietary habits. Countless factors influence our ways of eating. An important role is played by cultural, financial or geographic aspects. Various types of diets are known, such as basic, alternative, therapeutic, experimental and many more. Occasionally, new diets are proposed. They are based on, for example, including or excluding a certain dietary group from everyday consumption, a specific caloric balance or the content of basic nutrients—proteins, fats or carbohydrates. Regardless of the scientific basis for creating a given diet or the effectiveness of achieving its goal, the WHO pyramid of healthy eating remains a universal determinant of proper nutrition. According to its new version, plant products should form the basis of our diet and be consumed in the largest quantities.

Transcription factors are proteins that possess the ability to bind DNA in the promoter or enhancer sequence at a specific site or region, where they regulate the transcription process. Transcription factors are essentially classified by three different aspects: mechanism of action, regulatory function and sequence homology in their DNA-binding domains. Due to the fact that they help to "turn on" or "turn off" individual genes, they directly affect a number of parameters of cell functions and cellular metabolism. Thus, in a broader context, transcription factors affect the work of different organs, e.g., the liver, or entire systems, e.g., cardiovascular [1,2,3,4,5].

Five transcription factors play a crucial role in lipid and cholesterol metabolism: liver X receptor (LXR), peroxisome proliferator-activated receptor-α (PPAR-α), peroxisome proliferator-activated receptor-γ (PPAR-γ), CCAAT/enhancer binding protein α (C/EBPα) and sterol regulatory element-binding protein 1c (SREBP-1c). There are many groups of plant substances that influence the expression of these factors, and thus also the genes whose functioning they regulate. This impact was confirmed in numerous studies—both tissue and animal as well as human models. Among the most frequently mentioned compounds exhibiting such effects, the following are worth mentioning: polyphenols, phytoestrogens, soy proteins, mono- and polyunsaturated fatty acids, alkaloids, saponins, and also anthocyanins and iridoids. The latter two are the subject of our particular interest. Studies summarizing the current knowledge on the effect of, e.g., polyphenols or unsaturated fatty acids, on the transcription factors associated with lipid and cholesterol turnovers are quite common. However, to the best of our knowledge, there is no publication collecting data on this subject in the case of anthocyanins and iridoids. Therefore, we decided to assemble and systematize information on the impact of anthocyanins and iridoids on transcription factors crucial for lipid and cholesterol metabolism.

## 2. Major Transcription Factors Involved in Lipid and Cholesterol Metabolism

Replication, transcription and translation—at the cellular level—play a key role in the reproduction, development and maintenance of life processes in all organisms. The genome contains all information regarding the structure and function of bodily proteins. The process of protein formation is indirectly controlled by a specific group of proteins and nuclear receptors, described as transcription factors (TFs). The transcription process never enfolds the whole DNA strand, but only the fragment that encrypts a protein that is currently required by the body. This phenomenon is determined, among others, by transcription factors. Their task, apart from the transcription induction itself, is to direct the appropriate signal to the promoter site of the gene encoding the desired protein [6,7]. It was found that a dysfunction of transcriptional factors is related to a third of human developmental disorders. Moreover, TFs occur numerously among the group of oncogenes [3].

Transcription factors control initiation or elongation processes. Certain TFs may affect both of these processes. They typically bind cofactors—protein complexes that are associated with activation (coactivators) and repression (corepressors) but do not have DNA-binding capabilities of their own. Most TFs are thought to contribute to transcription initiation precisely by recruiting coactivators [2].

Transcription factors, as mentioned earlier, can be divided according to various criteria, but the simplest classification differentiates them into two basic groups: general transcription factors (GTFs) and specific transcription factors (STFs). GTFs, which always remain active, forming the pre-initiation complex, are responsible for inducing the transcription process. Six general factors can be distinguished: TFIIA, TFIIB, TFIID, TFIIE TFIIF, and TFIIH. In turn, specific factors accelerate or inhibit the transcription of target genes in response to signals sent to the cell nucleus. These factors are activated or suppressed depending on the demand for specific proteins [6,7,8].

STFs attach to the DNA fragment within the promoter, which leads to facilitating or hindering the recognition of the binding sequence by the pre-initiation complex. This conflation takes place by using small binding proteins, called either enhancers or silencers, i.e., short DNA sequences that are located even up to several thousand base pairs away from the promoter. The attachment of the transcription factor to the enhancer results in accelerated transcription, while silencers cause the opposite effect [2,9]. This is why STFs are a very engaging research target and therapeutically promising factors for many substances, including various food compounds.

To date, over 1000 various transcription factors have been described in humans [3]. Five of them play a key role in lipid and cholesterol metabolism: liver X receptor (LXR), peroxisome proliferator-activated receptor-α (PPAR-α), peroxisome proliferator-activated receptor-γ (PPAR-γ), CCAAT/enhancer binding protein α (C/EBPα) and sterol regulatory element-binding protein 1c (SREBP-1c).

Liver X receptor is a ligand-activated transcription factor of the nuclear receptor superfamily, playing a critical role in the regulation of the expression of major genes involved in cholesterol, lipid and glucose homeostasis. Two isoforms of liver X receptor can be distinguished: LXRα and LXRβ. It was proven that LXRs’ reverse the transportation of cholesterol to peripheral tissues and transport the excess cholesterol into the liver, reduce cholesterol absorption by intestinal epithelial cells and directly induce the expression of the essential transcription factor for lipid and cholesterol synthesis in the liver, sterol regulatory element-binding protein 1c [10,11]. One of the first direct LXR target genes identified was ATP-binding cassette transporter A1 (ABCA1). LXR activation leads to the robust upregulation of ABCA1 in macrophages, the intestine and in the liver, and the efflux of cholesterol to apolipoprotein A1, which results in the formation of high-density lipoproteins (HDL) [10,12]. LXRs are responsible for the stimulation of HDL synthesis [13]. 

Cholesterol is, above all, an essential constituent of the cell membrane but also represents a precursor for the synthesis of several hormones and bile acids that play significant roles in various physiological processes. Incorrect maintenance of cholesterol homeostasis, including absorption, biosynthesis, transmission and efflux, may contribute to severe disorders, such as atherosclerosis, metabolic syndrome, cardiovascular disease (CVD) or cholelithiasis [14]. Therefore, LXR as a cholesterol sensor play a critical role in its metabolism and protection against atherosclerosis [15,16,17]. It is worth highlighting that synthetic LXR agonists may exert undesirable effects on plasma and hepatic triglycerides through activating another transcriptional factor—hepatic sterol regulatory element-binding protein 1c [18]. For this reason, natural LXR agonists, which could be deprived of these side effects, are urgently needed. 

Peroxisome proliferator-activated receptors (PPARs) are a superfamily of nuclear receptors functioning as ligand-activated transcription factors. PPARs include PPAR-α, PPAR-δ and PPAR-γ subtypes. PPARs play a relevant role in the regulation of glucose and lipid metabolism. PPAR-α, highly expressed in the liver, is closely related to fatty acid β-oxidation in the liver and in brown adipose tissue and also modulates both acute and chronic inflammation [11,19]. To a lesser extent, it occurs in the cardiovascular system, muscles and kidneys. PPAR-α regulates the transcription of multiple genes, including acyl-CoA-oxidase, carnitine palmitoyl transferase (CPT) and several CYP4As [20]. PPAR-δ is expressed in almost all the tissues of the body. PPAR-γ is a major regulator expressed in fat tissue that promotes fat storage in white adipose tissue and also, to a lesser extent, in the liver, muscles and cardiovascular system [11]. In the case of overweight or obesity, white adipose tissue plays a pivotal role in the development of oxidative stress, inflammation and depletion of n-3 long-chain polyunsaturated fatty acids [21].

Two essential transcription factors that adjust adipocyte differentiation and regulate the expression of adipogenic and lipogenic genes are PPAR-γ and CCAAT/enhancer binding protein α (C/EBPα) [22]. CCAAT/enhancer binding protein α is a member of a family of six transcription factors, named from C/EBPα to C/EBPζ. They interact with the CCAAT (cytosine–cytosine–adenosine–adenosine–thymidine) sequence, present in several gene promoters. C/EBPs are pivotal in adipogenesis (C/EBPβ and C/EBPδ are early adipogenic transcription factors, while isoform α regulates the final stages of adipogenesis) and the development of osteoporosis. C/EBPα is one of the inductors of PPAR-γ expression [23,24]. Importantly, from a potential therapeutic perspective, the suppressor of PPAR-γ, C/EBPα, and also SREBP-1 expression, is AMP-activated protein kinase (AMPK) [25,26]. AMPK is a key factor controlling cellular energy homeostasis. It influences hepatic lipid metabolism through modulating the downstream acetyl-CoA carboxylase (ACC) and carnitine palmitoyl transferase-1 (CPT-1) pathway [27]. 

Another crucial transcription factor that enhances lipogenesis and adipogenesis is sterol regulatory element-binding protein 1c (SREBP-1c). There are two isoforms of sterol regulatory element-binding protein 1, SREBP-1a and SREBP-1c. SREBP-1a is expressed in the intestine and spleen, whereas SREBP-1c is mainly expressed in the liver, muscle and adipose tissue. SREBP-1c regulates genes required for glucose metabolism, fatty acid and lipid production and facilitates the storage of fatty acids as triglycerides [28,29,30]. Its expression is induced i.a. by insulin. A high-fat diet (HFD) is another key factor triggering the overexpression of both SREBP-1c and PPAR-γ in the liver and adipose tissue [31,32]. 

## 3. Impact of Anthocyanins

**Figure 1:**
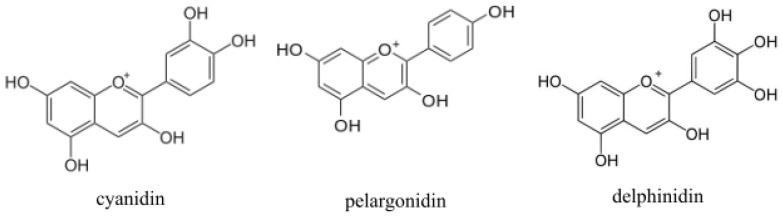
Chemical structures of main anthocyanins’ aglycons.

Anthocyanins are a group of natural substances that have garnered increasing interest in recent years. Anthocyanins are natural polyphenolic glycosidic phytopigments (mainly red, blue or purple), occurring primarily in fruits and flowers, that are biochemically related to flavonoids. Structurally, anthocyanins are aliphatic or aromatic three-ring compounds with one or more sugar molecules, and sometimes with a sugar attached aryl group. The colored aglycons are the anthocyanidins (usually cyanidin, pelargonidin or delphinidin) [33] (Figure 1). Fruits, which contain anthocyanins, are the sources of 90% of their habitual consumption [34]. Although anthocyanins are common in fruits, their daily intake by individual people in western countries is often relatively low [35]. The most common sources of these compounds are blueberries, blackberries, cranberries, acai berries, chokeberries, purple sweet potatoes, cherries, redcurrants or black soybeans. Anthocyanins possess potential anti-oxidative, antimicrobial, anti-inflammatory, anti-aging, lipid-lowering, anti-diabetic, anti-cancerous and anti-obesity bioactivities [36,37].

Anthocyanins suppress lipid accumulation in adipocytes due to the broad inhibition of the transcription factors regulating lipogenesis. This may partially explain the mechanism by which anthocyanins exert their anti-obesity effect. Anthocyanins may also lessen the process of triglyceride and lipid accumulation during adipocyte differentiation [38]. They reduce the gene and protein expression levels of lipogenic transcription factors such as liver X receptor α, sterol regulatory element-binding protein 1c, peroxisome proliferator-activated receptor-γ and CCAAT/enhancer binding protein α. A fortiori, target genes’ and proteins’ expression of these TFs is also distinctly suppressed by anthocyanins [38].

One of the most common anthocyanidins (aglicons of anthocyanins) is cyanidin. Cyanidin is natural compound abundant in fruits and vegetables, i.a. hawthorn, hibiscus or blueberry [33]. Jia et al. [13] showed that cyanidin is a direct ligand for both LXRα and LXRβ. Moreover, cyanidin affects all three PPAR subtypes, with the greatest affinity to PPAR-α [39]. Cyanidin-3-*O-β*-glucoside (C3G) activates liver X receptor (LXR)-ATP-binding cassette transporter-dependent cholesterol efflux [40,41] and phosphorylation of cellular AMPK in human hepatoma (HepG2) cells [27,42,43]. It was proven that C3G decreases the expression of adipogenic transcription factors involved in hepatic lipid metabolism, such as SREBP-1c, C/EBPα and PPAR-γ, both in the HepG2 cells and in the livers of supplemented mice [44]. The decreasing influence of anthocyanins on the gene expression of SREBP-1c and C/EBPα was also validated in other studies [43,45,46,47]. 

Nonetheless, the issue of anthocyanins’ effects on PPAR-α activation still requires more research. There have been reports that anthocyanins have a strong affinity and a significant impact on the expression of this receptor [39,41,45,48], while Rimando et al.’s [49] results on the hamster model stand in opposition to the above findings.

Anthocyanins have been reported to have beneficial effects on obesity and obesity-related metabolic disorders (e.g., insulin resistance and dyslipidemia). Anthocyanins elicited from, e.g., purple corn, sweet cherry, hibiscus or black soybean, in mice or 3T3-L1 preadipocyte models, prevented adipocyte differentiation, lipid accumulation and low-density lipoprotein (LDL) oxidation and protected against diet-induced hepatic steatosis by reducing PPAR-γ transcriptional activity [50,51,52,53]. A very abundant source of anthocyanins is the fruits of *Aronia melanocarpa* (AM). Park et al. [54] conducted an interesting AM study both on cells and mice. It appeared that AM extract significantly reduced free fatty acid-induced lipid droplet accumulation in vitro. Moreover, the synthesis of PPAR-γ2 was inhibited in vivo, the transcriptional activity of PPAR-γ2 was downregulated in vitro and the mRNA expression of PPAR-γ2 and its target genes, adipocyte protein 2 and lipoprotein lipase, was lowered both in vitro and in vivo.

However, in a recently conducted human study, berry supplementation triggered an inhibition of nuclear factor-kappa B (NF-κB)-dependent gene expression, accompanied by an enhancement in PPAR-γ expression. Nevertheless, an improvement in selected features of metabolic syndrome and related cardiovascular risk factors was observed [55]. Therefore, it can be hypothesized that stimulation or inhibition of PPAR-γ expression by anthocyanins may have both positive and negative consequences. Stimulation of this transcription factor may contribute to the reduction of inflammation while acting as a lipogenic, whereas the inhibition of PPAR-γ expression brings beneficial effects in the context of lipid homeostasis. Overall, it may lead to a partial prevention of obesity-induced atherosclerosis by attenuating inflammatory responses. It is also worth remembering that there are differences in fat metabolism between rodents and humans, which also may translate into disparities in the results of individual research models.

In 2019, Park et al. [37] confirmed most of the properties attributed to anthocyanins in earlier studies. They proved that another common anthocyanin—Delphinidin-3-*O-β*-glucoside (D3G)—significantly inhibited the accumulation of lipids in a dose-dependent manner without displaying cytotoxicity. In the 3T3-L1 adipocytes, D3G lowered the expression of major adipogenic and lipogenic transcription factors, i.e., PPAR-γ, SREBP-1 and C/EBPα. In addition, D3G enhanced the phosphorylation of AMPK and ACC—a fatty acid oxidation enzyme. These results once again supported the previously presented hypothesis that anthocyanins attenuate adipogenesis and promote lipid metabolism and are potential therapeutic agents in the treatment of obesity. Similar findings were obtained with a sole aglicon, delphinidin [56]. 

Anthocyanins represent also an interesting therapeutic option in diabetic nephropathy. Renal damage occurring in this disorder is mainly caused by oxidative stress and lipotoxicity. Db/db mice treated with anthocyanins show decreased albuminuria, ameliorated intra-renal lipid concentrations and improvement of glomerular matrix expansion and inflammation related to an increase in AMPK phosphorylation, upregulation of PPAR-α and PPAR-γ and decrease in SREBP-1 activity [48]. The studies describing the impact of anthocyanins on the discussed transcription factors are summarized in Table 1.

## 4. Impact of Iridoids

**Figure 2:**
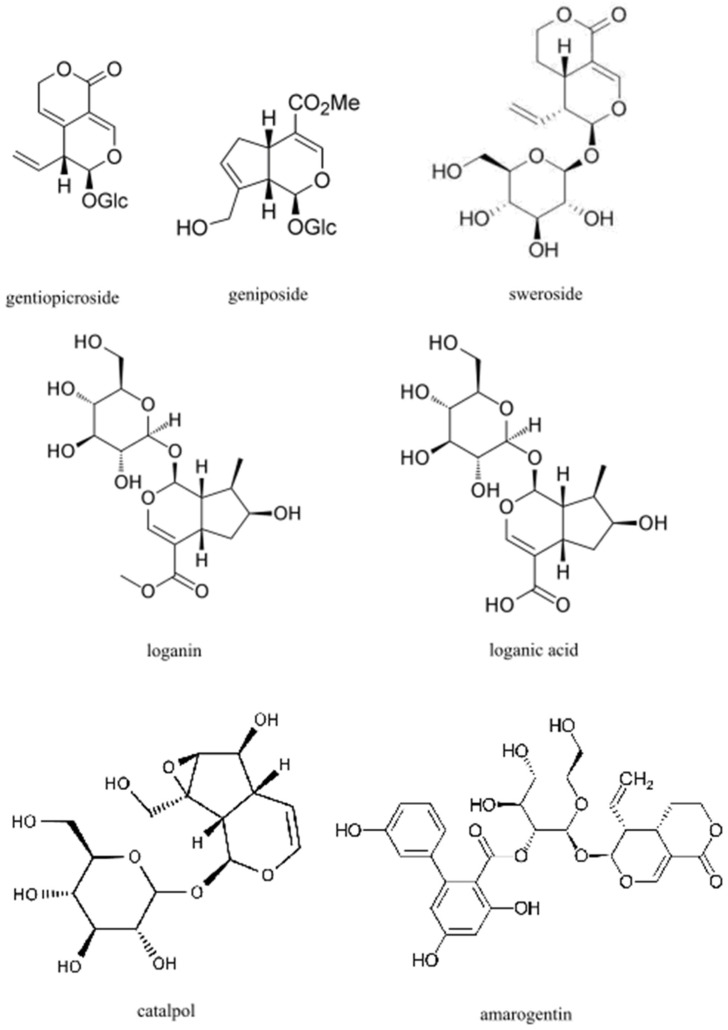
Chemical structures of main iridoids.

Iridoids are a large group of organic monoterpenoids, occurring in plants usually as glycosides with a glucose moiety attached to C-1 in the pyrene ring. The basic structural feature of iridoids is a bicyclic H-5/H-9β, β-cis-fusedcyclopentanopyran ring system. Iridoids are found in the green parts of plants, mainly in the leaves and young stems, and sometimes in fruits and sprouts. They are found in many plant families, e.g., *Apocynaceae*, *Gentianaceae*, *Lamiaceae*, *Loganiaceae*, *Rubiaceae*, *Scrophulariaceae* and *Verbenaceae.* Among the most commonly mentioned iridoids in the therapeutic context, the following stand out above all: gentiopicroside, geniposide, sweroside, loganin, loganic acid, catalpol and amarogentin [33] (Figure 2). In foods consumed in western diets, they are rarely present. However, they can be found in some raw fruits (e.g., olives, cornelian cherry, honeysuckle berries and cranberries) and in products derived from these fruits [57,58,59,60]. Iridoids possess potential cardiovascular, hypoglycemic, hypolipidemic, antihepatotoxic, choleretic, anti-inflammatory, antispasmodic, antitumor, antiviral, immunomodulatory and purgative bioactivities [61].

MicroRNAs are short, non-coding, single-stranded RNA molecules which have been reported to play a key role in the adjustment of lipid and lipoprotein metabolism. Increased levels of some miRNAs have been observed in the circulation in the course of hyperlipidemia [62]. It was proven that genipin upregulates the expression of miR-142a-5p, which binds to SREBP-1c and consequently leads to the inhibition of lipogenesis, which results in decreased body weight, lipid serum levels and hepatic lipid accumulation in HFD mice [63]. However, it is worth noting that there are a lot of miRNAs involved in fat metabolism, and some variants grow while others decline in the course of hyperlipidemia.

Zhu et al. [64] conducted an interesting study whose assumptions were based on providing HFD rats with three different doses of an iridoid fraction isolated from *Valeriana jatamansi.* Compared to the model group, a decrease in weight gain as well as a decrease in triglyceride and an increase in HDL-C serum levels were noticed regardless of the dose. All doses enhanced the expression of the PPAR-α receptor and reduced the expression of the SREBP-1c protein in the liver. In addition, depending on the dose, alterations in liver X receptor-α expression were observed. Hypolipidemic and antioxidant activity of iridoids through the stimulation of PPAR-α was noted also in different studies [36,65,66,67,68].

*Cornus alternifolia* (CA) is a tree that is common mainly in Eastern Asia and North America. Extracts of this plant have been used in traditional Chinese medicine as tonics, analgesics and diuretic drugs, while, in the United States, CA is grown as an ornamental plant. *Cornus alternifolia* leaves are a valuable source of iridoid compounds. It was shown that one of them—kaempferol-3-*O-β*-glucopyranoside—exhibited significant agonistic activities for PPAR-α, PPAR-γ and LXR dose-dependently in human hepatoma (HepG2) cells and Chinese hamster ovary cells (CHO) [69]. Several other iridoid substances isolated from CA leaves also influenced the expression of these transcription factors, but it is kaempferol-3-*O-β*-glucopyranoside which presents the most promising curative potential [69]. *Fraxinus* is a tree genus from which valuable quantities of iridoid compounds can also be obtained. *Fraxinus* compounds show significant inhibitory activity on adipocyte differentiation in the 3T3-L1 cells, reduce fat and triglyceride accumulation in differentiated 3T3-L1 cells without affecting cell viability and suppress the induction of C/EBPα, C/EBPβ and PPAR-γ transcription factors, concomitantly activating the PPAR-α receptor [70,71]. Similar effects were noticed in the case of iridoids of olive trees [72].

As with anthocyanins, iridoids, e.g., catalpol, may be a useful therapeutic option for the treatment of diabetes complications [73]. Gentiopicroside, a compound that belongs to the sub-group of seco-iridoids, ameliorated dyslipidemia and improved nerve blood flow through regulating the PPAR-γ/AMPK/ACC signal pathway in a diabetic peripheral neuropathy rat model [74]. Moreover, gentiopicroside in both acute and chronic alcohol-induced mouse hepatosteatosis (the latter stage of alcoholic liver disease, ALD) models mitigated the upregulation of SREBP-1 and downregulation of PPAR-α, among others, by activation of AMPK [75]. Alleviation of SREBP-1 expression was observed also in a nonalcoholic fatty liver disease model, namely by swertiamarin [76]. 

Iridoids and seco-iridoids such as catalpol, geniposide, harpagoside, loganin and oleuropein have been found to exhibit essential neuroprotective effects in Alzheimer’s disease (AD) and Parkinson’s disease (PD). One of the proposed mechanisms by which the process of neurogedeneration is slowed down is by increasing the PPAR-γ receptor expression, which contributes to the clearance of amyloid-β in the apolipoprotein E-mediated pathway in the brain [77].

For the prevention and treatment of obesity and dyslipidemia, a potentially useful iridoid compound is loganic acid (LA). Park et al. [78] demonstrated the antiadipogenic effects of LA in vitro and in vivo. Loganic acid treatment significantly decreased the adipocyte differentiation of 3T3-L1 preadipocytes in a dose-dependent manner and substantially lowered the expression of key adipogenesis transcription factors such as peroxisome proliferator-activated receptor-γ and CCAAT/enhancer-binding protein α. Moreover, a diminution of body weight gain, total fat increase, fatty hepatocyte deposition in the liver and adipocyte enlargement in the abdominal visceral fat tissues were noticed in an ovariectomy-induced obesity mice model compared to the comparative group. Loganic acid is also an important active ingredient found in cornelian cherry fruits. *Cornus mas* fruits, abundant in substantial amounts of both anthocyanins and iridoids, may exert positive additive or synergistic effects on dyslipidemia and atherosclerosis. In a rabbit model of diet-induced dyslipidemia and atherosclerosis, Sozański et al. [36] demonstrated that both anthocyanins and, to a lesser extent, loganic acid increased the expression of PPAR-α and PPAR-γ receptors in the liver and decreased intima thickness and the intima/media ratio in the thoracic aorta. The studies describing the impact of iridoids on the discussed transcription factors are summarized in Table 2. 

## 5. Future Perspectives

Improper nutrition is a compelling problem nowadays. The share of plant products in the diets of many people should be much more extensive. In this context, compounds with proven therapeutic effects on the body, such as anthocyanins and iridoids, may play an increasingly important role in the prevention and adjunctive or combined treatment of many human diseases. Many studies confirmed the curative effects and mechanisms of action of these groups of substances. Unfortunately, the majority of these studies were performed on animals, and first and foremost, so the paramount goal for the near future is to conduct more clinical studies to confirm the positive impact of anthocyanins and iridoids on the human body. New applications for the above-mentioned compounds should also be sought. Interestingly, most of the conducted research pertains to the influence of anthocyanins and iridoids on high-fat-diet models. It would certainly be a challenging option to consider a study, or a whole series, verifying the impact of these substances on multiple parameters in a low-fat diet.

## 6. Conclusions

**Figure 3:**
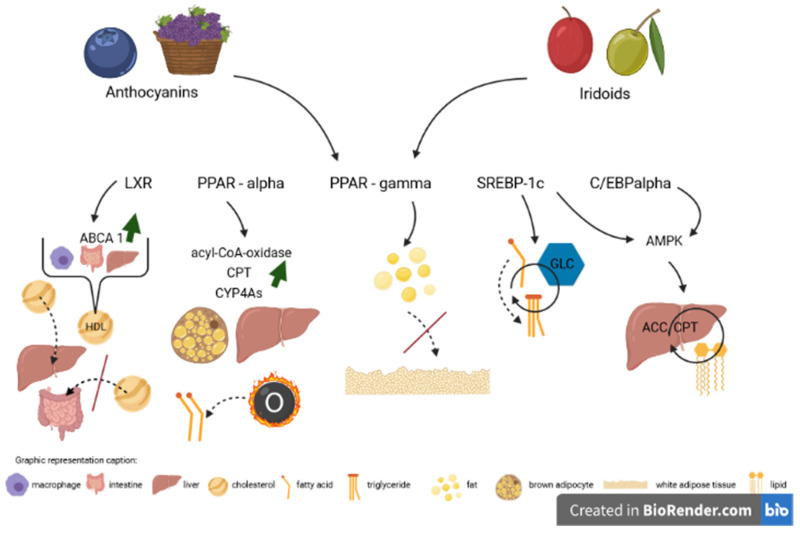
Influence of anthocyanins and iridoids on main transcription factors involved in cholesterol and lipid metabolism. Accessed on 28 May 2021.

In conclusion, the current knowledge clearly points to the benefits of consuming foods comprising anthocyanins and iridoids. Both groups of substances modulate the expression of crucial transcription factors in lipid and cholesterol homeostasis, i.e., liver X receptor, peroxisome proliferator-activated receptor-α, peroxisome proliferator-activated receptor-γ, CCAAT/enhancer binding protein α and sterol regulatory element-binding protein 1c and their target genes (Figure 3). Therefore, anthocyanins and iridoids present an essential therapeutic option in disorders proceeding with disturbances in lipid and cholesterol metabolism, such as obesity, atherosclerosis, diabetes, metabolic syndrome and many more.

## Figures and Tables

**Table 1:** List of studies describing the impact of anthocyanins on discussed transcription factors.

Authors and Date of Publication	Research Model	Compounds Used in Study	Observed Changes
Aboonabi et al., 2020 [55]	human	berry anthocyanin supplements	PPAR-γ 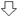
Chang et al., 2013 [43]	HepG2 cells	mulberry anthocyanin extract	SREBP-1c 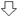 PPAR-α 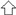
de Sousa et al., 2018 [45]	rats	extruded sorghum flour	SREBP-1c 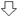 PPAR-α 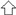
Du et al., 2015 [41]	HK-2 cells	cyanidin-3-*O-β*-glucoside, cyanidin	LXRα 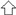 PPAR-α 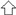
Fu et al., 2014 [40]	mice, mice mammary epithelial cells	cyanidin-3-*O-β*-glucoside	LXRα 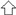
Hwang et al., 2011 [42]	mice	purple sweet potato anthocyanin fraction	SREBP-1c 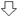
Jia et al., 2013 [39]	HepG2 cells, CHO-K1 cells	cyanidin	PPAR-α 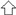 PPAR-δ 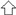 PPAR-γ 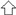
Jia et al., 2013 [13]	macrophages, hepatocytes	cyanidin	LXRα, LXRβ 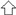 SREBP-1c 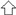
Kao et al., 2009 [52]	mouse macrophage J774A.1 cells	hibiscus anthocyanin extract	PPAR-γ 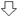
Khan et al., 2018 [47]	3T3-L1 cells	*C. kousa* anthocyanin ethanolic leaf extract	PPAR-γ 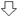 C/EBPα 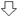
Kim et al., 2012 [53]	3T3-L1 cells	black soybean anthocyanin extract	PPAR-γ 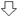
Koh et al., 2015 [48]	mice	*Seoritae* anthocyanin extract	PPAR-α 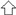 PPAR-γ 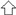 SREBP-1c 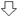
Lee et al., 2014 [38]	3T3-L1 cells	grape anthocyanin isolate	LXRα 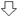 PPAR-γ 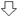 C/EBPα 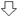 SREBP-1c 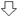
Luna-Vital et al., 2017 [50]	3T3-L1 cells	purple corn pericarp anthocyanin extract, pure anthocyanins	PPAR-γ 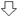
Park et al., 2015 [46]	rats	unfermented and fermented black carrot extract	SREBP-1c 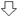 PPAR-α 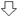
Park et al., 2017 [54]	mice, FL83B cells	*A. melanocarpa* spray-dried ethanol extract	PPAR-γ 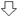
Park et al., 2019 [44]	HepG2 cells	honeyberry extract	SREBP-1c 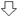 PPAR-γ 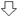 C/EBPα 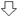 PPAR-α 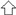
Park et al., 2019 [37]	3T3-L1 cells, primary white adipocytes	delphinidin-3-*O*-*β*-glucoside	PPAR-γ 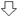 C/EBPα 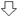 SREBP-1c 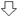
Rahman et al., 2016 [56]	3T3-L1 cells	delphinidin	PPAR-γ 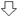 C/EBP 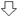
Rimando et al., 2016 [49]	hamsters	blueberry peel extract	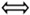 PPAR-α
Song et al., 2016 [51]	mice	sweet cherry anthocyanins	PPAR-γ 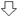
Sozański et al., 2014 [12]	rabbits	cornelian cherry fruits lyophilisate	PPAR-α 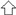
Sozański et al., 2016 [36]	rabbits	mixture of anthocyanins	PPAR-α 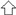 PPAR-γ 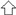

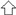
— up regulation, 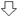
—down regulation, 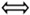
—unchanged.

**Table 2:** List of studies describing the impact of iridoids on discussed transcription factors.

Authors and Date of Publication	Research Model	Compounds Used in Study	Observed Changes
Bai et al., 2010 [71]	3T3-L1 cells	aqueous extract and compounds isolated from the seeds of *F. excelsior*	PPAR-α 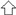
Choi et al., 2011 [70]	3T3-L1 cells	hydroxyframoside B	C/EBPα 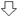 C/EBPβ 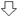 PPAR-γ 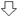
Drira et al., 2011 [72]	3T3-L1 cells	oleuropein	PPAR-γ 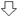 C/EBPα 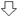 SREBP-1c 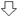
He et al., 2012 [69]	HepG2 cells, CHO cells	leaf extract of *C. alternifolia*, incl. Kaempferol-3-*O-β*-glucopyranoside	PPAR-α 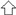 PPAR-γ 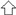 LXRα 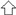
Li et al., 2018 [75]	mice, HepG2 cells, macrophages	gentiopicroside	SREBP-1c 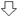 PPAR-α 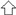
Lu et al., 2018 [74]	rats	gentiopicroside	PPAR-γ 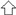
Ma et al., 2011 [66]	rats	geniposide	PPAR-α 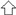
Malliou et al., 2018 [65]	mice	oleuropein	PPAR-α 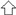
Park et al., 2018 [78]	3T3-L1 cells, mice	loganic acid	PPAR-γ 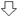 C/EBPα 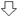
Patel et al., 2016 [67]	HepG2 cells	swertiamarin	SREBP-1c 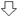 PPAR-α 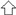
Sozański et al., 2014 [12]	rabbits	cornelian cherry fruits lyophilisate	PPAR-α 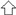
Sozański et al., 2016 [36]	rabbits	loganic acid	PPAR-α 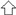 PPAR-γ 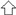
Yang et al., 2019 [76]	mice	swertiamarin	SREBP-1c 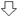
Yang et al., 2020 [68]	mice	sweroside	PPAR-α 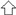
Zhong et al., 2018 [63]	mice, primary hepatocytes	genipin	SREBP-1c 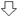
Zhu et al., 2016 [64]	rats	iridoids rich fraction in *V. jatamansi*	LXRα 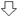 SREBP-1c 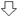 PPAR-α 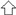

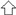
—up regulation, 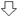
—down regulation.

## Abbreviations

ABCA1	ATP-binding cassette transporter A1
HDL	high-density lipoprotein
acyl-CoA-oxidase	acyl coenzyme A oxidase
CPT	carnitine palmitoyl transferase
CYP4As	cytochrome P450 4A subfamily enzymes
GLC	glucose
AMPK	AMP-activated protein kinase
ACC	acetyl coenzyme A carboxylase
O	β-oxidation
